# Effect of docetaxel administration on fluid dynamics in mice

**DOI:** 10.20407/fmj.2024-023

**Published:** 2024-12-27

**Authors:** Ayana Mawaki, Masushi Kohta, Aya Yoshimura, Toshio Nakatani, Shizuko Nagao, Junko Sugama

**Affiliations:** 1 Graduate School of Health Sciences, Fujita Health University, Toyoake, Aichi, Japan; 2 Department of Integrated Health Sciences, Nagoya University Graduate School of Medicine, Nagoya, Aichi, Japan; 3 Research Center for Implementation Nursing Science Initiative, Fujita Health University, Toyoake, Aichi, Japan; 4 Advanced Medical Research Center for Animal Models of Human Diseases, Fujita Health University, Toyoake, Aichi, Japan; 5 Department of Nutrition, Faculty of Health Science, Hokuriku Gakuin University, Kanazawa, Ishikawa, Japan

**Keywords:** Docetaxel, Edema formation, Fluid dynamics, Mice

## Abstract

**Objectives:**

The taxane chemotherapeutic agent docetaxel has been used as a therapy for various cancers. Some patients receiving docetaxel develop serious problems with fluid retention, which leads to peripheral edema formation, reducing the patient’s quality of life. This study investigated the effect of docetaxel administration on fluid dynamics in mice as a step toward developing advanced preventive measures in nursing.

**Methods:**

Mice were administered 10 mg/kg/day of docetaxel intravenously for 5 days as the intervention group or with normal saline as the control group. To investigate fluid dynamics on day 5, the leakage of blood plasma, interstitial fluid volume, and fluid transportation capacity into lymph vessels were evaluated and compared between the two groups.

**Results:**

The Miles assay with Evans Blue, an albumin-binding dye, revealed that leakage of blood plasma was significantly increased in the control group compared with the intervention group (p<0.01). Results of the interstitial fluid volume and fluid transportation capacity were similar between the two groups, but the fluid transportation capacity tended to be higher in the intervention group.

**Conclusions:**

Docetaxel administration in our mouse model caused the leakage of blood plasma without proteins from the blood vessels into the interstitial tissues, which appeared at the initial stage of edema formation. This model might be useful for assessing the leakage of blood plasma and, subsequently, the development of preventive measures against edema formation.

## Introduction

Docetaxel (DTX), which belongs to the class of taxane neoplastic agents, was reported to be one of the most effective anticancer agents. However, its clinical benefit is accompanied by intolerable adverse effects, including fatigue, hair loss, numbness, fluid retention syndrome, and edema formation.^1–3^ Edema is generally defined as excessive fluid retention in the interstitial space beyond the physiological compensatory capacity.^4^ The incidence rate of DTX-related edema formation varies, ranging from 30% to 84%,^5–7^ because the evaluation of DTX-related edema formation is often performed by a visual inspection as a routine clinical examination. The most predisposed site for edema formation is the lower limbs. Unfortunately, edema formation in the lower limbs results in poor appearance, affects patient quality of life, and then potentially causes the development of lymphedema and skin hardening;^8–10^ therefore, there is a need to develop preventive measures against edema formation caused by DTX administration.

Understanding the mechanism by which edema is formed in the lower limbs by DTX administration is important to develop preventive measures in nursing science. To the best of our knowledge, two animal models of docetaxel-induced edema have been reported; Brønstad et al.^11^ generated a DTX-related edema formation model by the subcutaneous administration of DTX to the foot skin of rats, and they evaluated albumin extravasation using ^125^I-labeled albumin. However, this model is not appropriate for assessing DTX-related edema because DTX is repeatedly administered intravenously in clinical practice. To investigate the toxicity of DTX nanoparticle powder, Choi et al.^12^ prepared a subcutaneous tumor mouse model where DTX was injected intravenously once a day for five consecutive days, and paw edema was measured by a plethysmometer, designed to precisely measure paw volume and swelling in rodents for inflammation research. However, they only assessed paw volume, which was insufficient to examine the mechanisms related to edema formation.

Imakata et al. observed skin tissue images using an ultrasound device and found that fluid retention inside the tissue was generated before swelling with pitting edema.^13^ We focused on changes in vascular permeability, fluid retention in tissues, and interstitial fluid flow in lymph vessels caused by DTX administration that were expected to occur before the clinical symptoms of edema developed. The principal mechanism underlying edema formation is thought to be increased vascular endothelial cell permeability, resulting in the extravasation of plasma albumin into the surrounding tissues.^14^ The severity of this reaction is proportional to the cumulative dose of the drug administered. Considering that edema is swelling caused by the expansion of interstitial fluid volume in tissues, DTX administration might inhibit the fluid drainage pathway in lymphatic vessels. However, there have been no studies on the effect of DTX administration on vascular permeability, volume of fluid retention, and ability of fluid drainage in parallel.

We hypothesized that repeated intravenous DTX administration would increase vascular permeability via vascular endothelial cell injury, leading to increased interstitial fluid retention and inhibited interstitial fluid transportation to lymphatic vessels. In this study, a mouse model was used to evaluate the effect of DTX administration on the leakage of blood plasma through capillary walls, interstitial fluid volume increase, and fluid transportation capacity into lymph vessels in parallel. This study is the first step in clarifying the mechanism of action involved in edema formation by DTX injection. This study investigated the effect of DTX administration on fluid dynamics in mice as a step toward developing advanced preventive measures against edema formation in nursing.

## Materials and methods

### Animals

Animal strain selection was conducted in accordance with previous studies.^12^ Male ICR mice at 4 weeks of age were purchased from Japan SLC, Inc. (Shizuoka, Japan) and given free access to standard food and water under specific pathogen-free conditions in individual cages. A total of 34 mice were used for this experiment. Measurements of mouse body weight and food intake were conducted at 13:00 each day throughout this study. For each mouse, the average daily food intake was calculated by weighing the remaining food per day in a cage and dividing this by the number of mice in the cage. All animal procedures were approved by the Institutional Animal Care and Use Committee at Fujita Health University (approval number: AP22138-MD3).

### Evaluation of vascular permeability (Miles assay)

Ten mice were randomly divided into two groups and intravenously injected with 10 mg/kg of DTX (Wantaxotere; Sanofi, Paris, France) as the intervention group (n=5) or normal saline solution (Otsuka Pharmaceutical Co. Ltd., Tokyo, Japan) as the control group (n=5). The mice were injected five consecutive times in the lateral tail vein every day. The Miles assay, which is commonly used for quantifying vascular permeability, was used to assess DTX-induced vascular leakage in mice.^15,16^ In this procedure, Evans Blue (EB) dye was injected into the circulation, where it binds tightly to albumin. The extravasation of EB-bound albumin is used as an indicator of increased endothelial leakage in response to an inflammatory stimulus. Therefore, the amount of EB in the dermis is proportional to the amount of albumin in the dermis. One hour after DTX or normal saline was injected on day 5, the mice were injected with 0.2 mL 0.5% w/v EB (Fujifilm Wako Pure Chemical Corporation, Osaka, Japan) dissolved in normal saline via a lateral tail injection. Thirty minutes after the intravenous injection of EB, skin tissue samples at the torso and hind limb were collected, and EB dye was extracted with formamide (Fujifilm Wako Pure Chemical Corporation) for 24–48 h at 55°C. The extracted EB solution was centrifuged at 10,000×g and 4°C for 40 min. The amount of EB in each sample was determined by measuring the absorbance at 620 nm using a microplate reader (Multiskan FC, Thermo Fisher Scientific, Tokyo, Japan), and results were expressed as EB dye amount (μg) per sample weight (g) of the skin, with quantification against a standard curve.

### Interstitial fluid volume measurement

Twelve mice were randomly divided into intervention (n=6) and control groups (n=6). Before the DTX or normal saline injection on day 5, the whole-body composition of each mouse was measured using a Bioimpedance Spectroscopy device (ImpediVet™, ImpediMed Limited, QLD, Australia). The bioimpedance method is an indirect calculation of body composition that involves passing a weak electric current through the body and measuring its electrical resistance. Mice were anesthetized with 2%–3% isoflurane inhalation, then placed flat on their abdomen on a non-conductive surface with their fore and hind limbs positioned adjacent to their bodies. The tips of four 25G×25 mm needles were inserted under the skin on the dorsal midline of the mouse, between the two ears, 10 mm nasally from both ears, at the junction of the sacrum and tail, and 10 mm caudally from the junction of the sacrum and tailbone, where the measurement probes were attached. The probe insertion technique was in accordance with the manufacturer’s protocol. The weight of the mice and the distance between the measurement probes were input into the measuring machine, and the body composition was calculated from the magnitude of the electrical resistance of the weak current that flowed between the probes. The extracellular fluid volume per body weight was calculated and used for analysis.

### Interstitial fluid transportation capacity

Twelve mice were randomly divided into intervention (n=6) and control groups (n=6). The mice were fed an alfalfa-free diet to minimize autofluorescence and improve imaging clarity. Interstitial fluid transportation capacity was monitored using fluorescence imaging on a Lago X system (Spectral Instruments Imaging, Tucson, Arizona). In this procedure, indocyanine green (ICG) is stabilized by binding to albumin and is then injected subcutaneously. Mice were then placed into a dark box with a highly sensitive and cooled camera attached. The camera sensor was exposed for extended periods to capture fluorescence from the ICG in the mice. The acquired image was an integrated intensity map representing the amount of fluorescence of ICG transportation from tissues to lymph vessels.

In this study, mice were anesthetized by the inhalation of 2%–3% isoflurane and 5 μL of ICG (Diagnogreen for injection; Daiichi Sankyo Company Limited, Tokyo, Japan) prepared at 5 mg/mL was injected subcutaneously around the knee socket 10 mm above the heel of the right hind limb. ICG that had transferred to the lymph vessels was observed from the right inguinal lymph node toward the right axillary lymph node. The interstitial fluid transportation capacity was assessed by drawing a region of interest around the fluorescence intensity in the right axillary lymph node in the fluorescence images and recording the intensity value at 5, 10, and 15 minutes after ICG injection. The imaging parameters were: excitation wavelength=745 nm, emission wavelength=850 nm, exposure time=2s, binning value=8, and field of view=15. Fluorescence imaging was analyzed using Aura Imaging Software version 4.0.8 (Spectral Instruments Imaging) and presented as photons/s in regions of interest.

### Statistical analysis

Data were analyzed using IBM SPSS version 29.0.1.0. All data were continuous variables and expressed as the mean±standard deviations. Changes in the body weight and food intake of mice were compared by two-way analysis of variance, and the remaining factors were analyzed using an unpaired t-test to compare differences between intervention and control groups. In all cases, p<0.05 was considered statistically significant.

## Results

### Changes in body weight and food intake

**Figure 1:**
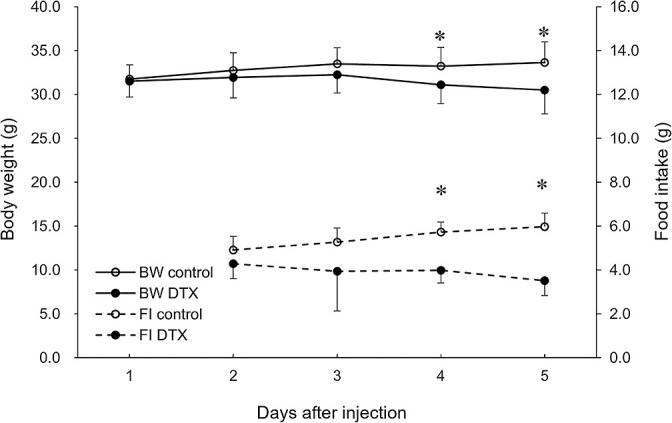
Effect of docetaxel administration on changes in body weight and food intake of normal mice. Two-way repeated measures analysis of variance for body weight and food intake indicated statistically significant differences between the intervention and control groups (n=17, p<0.001). *p<0.05 by *t*-test. Abbreviations: DTX, docetaxel. BW, body weight. FI, food intake.

The body weights of mice used in this study were measured every day throughout the study period. Average body weight reduction was observed in the intervention group but not in the control group. As shown in Figure 1, there were significant differences between the two groups on days 4 and 5 (p<0.001). Statistically significant differences in the body weight of mice on day 5 (30.52±2.83 g in the intervention group vs 33.65±2.41 g in the control group; p<0.001) and mean daily food intake (3.93±0.73 g in the intervention group vs 5.47±0.36 g in the control group; p<0.001) are shown in Figure 1.

### Evaluation of vascular permeability (Miles assay)

**Figure 2:**
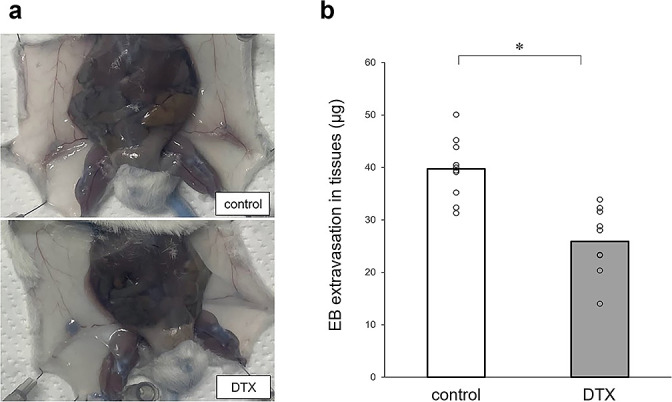
Vascular permeability. (a) Photograph of a vertical incision in a mouse made with scissors from the lower abdomen to the chest. The skin of both flanks was peeled off to expose the inner dermis. Blue areas would be observed on the skin in the event of Evans blue leakage, but there was no noticeable leakage of Evans blue into the skin in either group. (b) Evans-blue dye extravasation in skin biopsies (*p<0.05). Abbreviations: EB, Evans blue. DTX, docetaxel.

Figure 2a shows the visual observation of skin tissues after completing the EB injection. There was no noticeable leakage of EB into the skin in either group. Figure 2b shows a significant difference in dermal EB extravasation in skin biopsies from the intervention and control groups (25.87±6.16 μg vs 39.71±6.16 μg, respectively; p<0.01). This indicates the amount of albumin in the skin biopsy in the intervention group was lower than that in the control group.

### Interstitial fluid volume measurement

**Figure 3:**
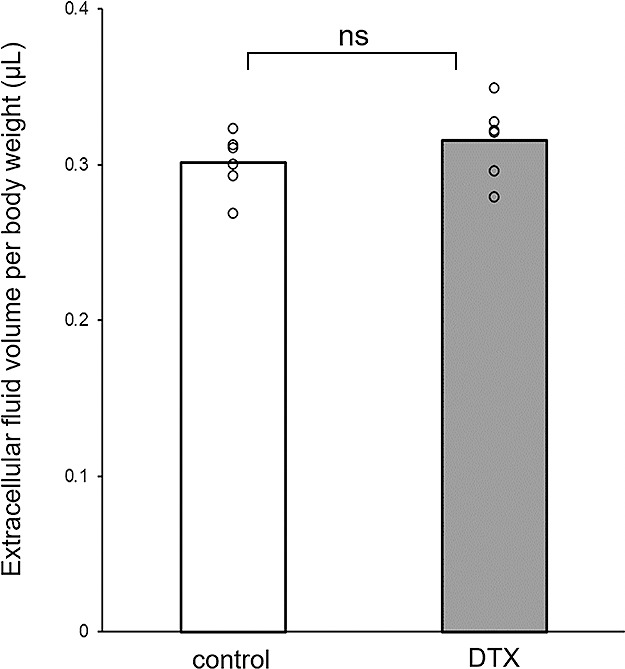
Interstitial fluid volume measured by bioimpedance spectroscopy. No statistically significant differences between the intervention and control groups were observed.

As shown in Figure 3, the interstitial fluid volume on day 5 was 0.32±0.03 μL and 0.30±0.02 μL in the intervention and control groups, respectively, with no significant difference between the two groups (p=0.288).

### Interstitial fluid transportation capacity

**Figure 4:**
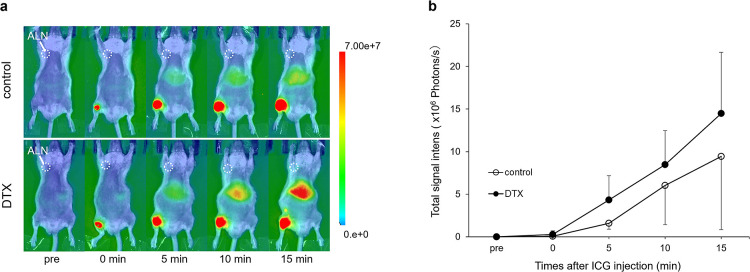
Fluorescence imaging test results. (a) Representative fluorescence images from normal mice obtained at different time points after the subcutaneous injection of ICG near the right knee fossa. ICG fluorescence intensity was evaluated around the right axillary lymph node (white circle). Immediately after ICG injection, a high fluorescence signal was observed in both groups at the injection site in the right hindlimb (red), but not around the right axilla; 15 minutes later, the fluorescence signal was increased in the right axilla in the DTX group (green). (b) Quantification of the fluorescence intensity in the right axilla is shown in (a). No statistically significant differences in the intensity values between the intervention and control groups were observed, but the intensity values in the intervention group were higher (p=0.065; after 5 min, p=0.387; after 10 min, p=0.339; after 15 min). Abbreviations: ALN, axillary lymph node. ICG, indocyanine green.

Figure 4a shows the accumulation of ICG in the right inguinal lymph node via the lymph vessels, and the intensity value was increased in the right axillary lymph node over time as ICG was transported through the lymph vessels. The intensity values for the intervention and control groups were 4.3±2.9 million photons/s and 1.6±0.7 million photons/s at 5 minutes, 8.5±4.0 million photons/s and 6.0±4.6 million photons/s at 10 minutes, and 14.5±7.1 million photons/s and 9.4±8.6 million photons/s at 15 minutes after ICG injection. The intensity value at each time point did not differ between the two groups; however, the intensity values in the intervention group were higher than in the controls (p=0.065 after 5 min, p=0.387; after 10 min, p=0.339; after 15 min, Figure 4b).

## Discussion

The development of edema in individuals receiving anticancer therapy negatively affects their quality of life. Promoting new preventive strategies against edema formation is essential for effective nursing care. However, little is known about interstitial fluid dynamics during the clinical course of edema formation caused by DTX administration, and there remains a lack of information on when and what kind of preventive care should be provided to patients. In this study, we investigated the effect of DTX administration on fluid dynamics, including vascular permeability, interstitial-fluid volume, and fluid transportation capacity, in parallel using normal mice. Our test results revealed the leakage of blood plasma without proteins from the blood vessels into the interstitial tissues in mice administered DTX. Therefore, this model might be useful for assessing the leakage of blood plasma and the development of preventive measures at the early stage of edema formation.

The most significant finding of this study was that EB vascular leakage in the dermis was higher in the control group compared with the intervention group (p<0.05; Figure 2b), which might have been related to the reduction of albumin levels in the blood caused by DTX and the increase of blood plasma. In addition, the intervention group exhibited higher interstitial fluid transportation capacity because excess interstitial fluid was collected and returned to the blood. This finding suggests that DTX administration in our mouse model increased the interstitial fluid volume, which appears at the initial stage of edema formation.

Regarding vascular permeability, we hypothesized that the repeated intravenous administration of DTX would increase vascular permeability by damaging vascular endothelial cells; however, our results did not confirm the hypothesis. This might be explained by a previous paper published by Semb et al.,^17^ which measured the amount of plasma and interstitial colloid osmotic pressures in patients who received DTX. In that study, the mechanism of action related to edema formation was as follows: 1) the amount of plasma was increased, which decreased the colloid-osmotic pressure during the early time course of treatments; 2) the plasma penetrated from blood vessels into the interstitial tissue; 3) water retention occurred in the interstitial tissue; and 4) high-molecular weight plasma proteins leaked into the interstitial tissue. Increased plasma volume leads to the relative reduction of serum albumin levels. Our vascular permeability testing may have been performed at a time when plasma without proteins had penetrated from blood vessels into the interstitium after DTX administration (from point 1 to point 2 as described above by Semb et al.). Compared with the control group, the intervention group had an increased plasma volume in blood vessels, a relatively decreased serum albumin concentration, and a lower amount of albumin-bound EB dye in the tissues.

Regarding the interstitial fluid transportation capacity, we hypothesized that fluid transportation from interstitial areas to lymph vessels was inhibited by DTX because of the generation of edema formation caused by increased vascular permeability and fluid retention in the interstitial tissues. However, our results were not concordant with the hypothesis. Béhar et al.^14^ investigated the relationship between cumulative DTX doses and the development of fluid retention by capillary filtration test analysis. Their results showed that a two-step process was required for fluid retention, with progressive congestion of the interstitial space by proteins and water, followed by insufficient lymphatic drainage. This suggests that lymphatic drainage occurred during the phase of the retention of water lacking plasma proteins such as albumin. Moreover, given the balancing point where the equilibrium interstitial inflow is equal to the equilibrium interstitial outflow, the lymphatic transport capacity would have been higher than in the normal state (Figure 4b). Therefore, the present model represents the early stage of edema formation and might be useful for developing methods to prevent early edema formation.

Future studies should develop a revised mice model of DTX administration to increase the leakage of blood plasma from the blood vessels into the interstitium. In the present study, it was difficult to induce vascular endothelial cell damage and the resulting adequate increase in the leakage of blood plasma via vascular permeability. Vascular permeability around tumor blood vessels was reported to be higher than around normal blood vessels.^18^ Tumor endothelial cells that line tumor blood vessels differ from normal endothelial cells in many aspects. Tumor endothelial cells are present as irregular monolayers with a higher expression of proangiogenic factors, and impaired endothelial barrier function compared with their normal counterparts. The basement membrane thickness of tumor blood vessels is uneven, and the association between pericytes and tumor endothelial cells is weak, leading to vascular leakiness. Thus, using a tumor mouse model with vascular complications rather than normal mice might be a more appropriate model to achieve enhanced vascular permeability by DTX injection.

In routine clinical examinations, DTX-related edema is often evaluated subjectively, and limited quantitative evaluation is provided. In the present study, the amount of interstitial fluid retention was assessed by the bioimpedance measurement technique, and we found that the intervention group tended to have higher interstitial fluid retention compared with the control group. The bioimpedance measurement technique is easy to perform and might be acceptable as an indicator of the early formation of edema in clinical practice.

This study had two limitations. The first was that we could not determine when the leakage of plasma from blood vessels into the interstitium was initiated because this study focused on a comparison of test results between the intervention and control groups on day five after DTX administration. To prevent edema formation at the earliest possible time, future studies should clarify the specific time point at which the leakage of plasma is initiated after DTX administration. Next, the validity of our mouse model could not be fully explained from our test results, because differences in changes in mouse body weight were observed between the intervention and control groups. The toxic effects of intravenously injected DTX on ICR mice included body weight loss, which is a common side effect of DTX treatment. Nonetheless, we think that our test results will enhance our understanding of the effects of DTX administration on fluid dynamics in mice, leading to the development of novel nursing technology to enhance the quality of life of patients undergoing anticancer therapy.

In conclusion, DTX administration to normal mice caused the leakage of blood plasma without proteins from the blood vessels into the interstitial tissues at the initial stage of edema formation. This model might be useful for assessing blood plasma leakage and developing preventive measures against early edema formation.
